# The relation between acute changes in the systemic inflammatory response and circulating thiamine and magnesium concentrations after elective knee arthroplasty

**DOI:** 10.1038/s41598-021-90591-y

**Published:** 2021-05-28

**Authors:** Donogh Maguire, Anthony Catchpole, Owen Sheerins, Dinesh Talwar, Alana Burns, Mark Blyth, Andrew Shaw, Bryn Jones, Colin Drury, Johann Harten, Innes Smith, Donald C. McMillan

**Affiliations:** 1 Emergency Department, Glasgow Royal Infirmary, 84 Castle Street, Glasgow, G4 0SF UK; 2 The Scottish Trace Element and Micronutrient Diagnostic and Reference Laboratory (STEMDRL), Department of Biochemistry, Macewen Building, Glasgow Royal Infirmary, Glasgow, G4 0SF UK; 3 Department of Orthopedic Surgery, Glasgow Royal Infirmary, Glasgow, G4 0SF UK; 4 Department of Anesthesia, Queen Elizabeth University Hospital, 1345 Govan Rd, Glasgow, G51 4TF UK; 5 Department of Orthopedic Surgery, Queen Elizabeth University Hospital, 1345 Govan Rd, Glasgow, G51 4TF UK; 6 Academic Unit of Surgery, School of Medicine, University of Glasgow, New Lister Building, Glasgow Royal Infirmary, 8-16 Alexandra Parade, Glasgow, G31 2ER UK

**Keywords:** Biochemistry, Immunology

## Abstract

Thiamine diphosphate (TDP) and magnesium are co-factors for key enzymes in human intermediary metabolism. However, their role in the systemic inflammatory response (SIR) is not clear. Therefore, the aim of the present study was to examine the relation between acute changes in the SIR and thiamine and magnesium dependent enzyme activity in patients undergoing elective knee arthroplasty (a standard reproducible surgical injury in apparently healthy individuals). Patients (n = 35) who underwent elective total knee arthroplasty had venous blood samples collected pre- and post-operatively for 3 days, for measurement of whole blood TDP, serum and erythrocyte magnesium, erythrocyte transketolase activity (ETKA), lactate dehydrogenase (LDH), glucose and lactate concentrations. Pre-operatively, TDP concentrations, erythrocyte magnesium concentrations, ETKA and plasma glucose were within normal limits for all patients. In contrast, 5 patients (14%) had low serum magnesium concentrations (< 0.75 mmol/L). On post-operative day1, both TDP concentrations (p < 0.001) and basal ETKA (p < 0.05) increased and serum magnesium concentrations decreased (p < 0.001). Erythrocyte magnesium concentrations correlated with serum magnesium concentrations (r_s_ = 0.338, p < 0.05) and remained constant during SIR. Post-operatively 14 patients (40%) had low serum magnesium concentrations. On day1 serum magnesium concentrations were directly associated with LDH (p < 0.05), WCC (p < 0.05) and neutrophils (p < 0.01). Whole blood TDP and basal ETKA increased while serum magnesium concentrations decreased, indicating increased requirement for thiamine and magnesium dependent enzyme activity during SIR. Therefore, thiamine and magnesium represent potentially modifiable therapeutic targets that may modulate the host inflammatory response. Erythrocyte magnesium concentrations are likely to be reliable measures of status, whereas serum magnesium concentrations and whole blood TDP may not.

**ClinicalTrials.gov**: NCT03554668.

## Introduction

In 2012, the National Research Council reported that dietary deficiency of vitamins and minerals affected > 80% of Americans^1,2^. Similarly, the NHANES III study reported that multi-nutrient deficiencies were more prevalent in those with a BMI in the obese range than in the normal population^3,4^.

Thiamine (vitamin B1) is an essential micronutrient that is required for optimal energy metabolism^5^. A healthy adult human has approximately a 3-week reserve of thiamine in the body. However, these reserves may be rapidly depleted in times of sustained physiological stress (e.g. post-surgery or during systemic infection)^6–11^. Nonetheless, while it is recognised that plasma measurements of thiamine status may be perturbed by the systemic inflammatory response, the direct measurement of thiamine diphosphate in red blood cells (erythrocytes) is known to reflect nutritional status and is not perturbed by the systemic inflammatory response^12–14^.

It is of interest that thiamine requires magnesium for conversion to its active form, thiamine diphosphate (TDP)^15^, and TDP requires magnesium to act as a co-factor for thiamine dependent enzymes^5^. Although this relationship has been well-understood biochemically for decades, the potential clinical relevance of such a relationship has received little attention to date^16,17^.

Magnesium is the second most abundant intracellular cation, with approximately 99% of total body magnesium localized to the intracellular compartment in bone and soft tissue, and only approximately 1% localized in the circulation^18^. There is an increasing awareness that magnesium plays an important role in energy metabolism^5^, cardiovascular health^19^, calcium homeostasis^20^, oxidative resilience and genomic stability^21^. Furthermore, low circulating magnesium concentrations have been implicated in increased prevalence of liver disease^22^ and significantly higher one year mortality among alcohol use disorder patients^23^. Accurate quantification of total body magnesium is challenging, as magnesium is predominantly located within intra-cellular compartments, while circulating serum magnesium is approximately 30% bound to proteins (mainly albumin) and it is reported that free measurable magnesium may be perturbed by the inflammatory response^18^.

There is now good evidence indicating that serum concentrations of other bivalent cations, including selenium and zinc, decrease transiently after a significant and reproducible inflammatory insult^24^. These measurements, therefore, may not be reliable indicators of selenium or zinc status in the context of significant activation of the systemic inflammatory response. It is therefore of interest that the inter-relationship between thiamine status and magnesium status, and the effect of the systemic inflammatory response on serum magnesium concentrations and functional markers of thiamine status has not been well delineated to date.

Therefore, the aim of the present study was to examine the relation between acute changes in the systemic inflammatory response and thiamine and magnesium dependent enzyme activity in patients undergoing elective knee arthroplasty.

## Patients and study design

The study protocol was approved by the East of England—Cambridgeshire and Hertfordshire Research Ethics Committee (17/EE/0270) and registered with clinicaltrials.gov (NCT03554668) (13/06/2018).

Patients who underwent elective primary total knee replacement surgery for osteoarthritis were considered eligible for study. Those patients with pre-operative CRP > 10 mg/L and reduced creatinine clearance (eGFR < 60 mL/ min/1.73 m^2^) prior to or following surgery were excluded from the study. In addition, patients who received thiamine/ magnesium supplementation during the study period were also excluded from the study.

Baseline demographic characteristics—including age, body mass index (BMI), and medical and drug history (including magnesium and thiamine supplementation), were recorded. Surgical procedures, tourniquet times, anesthetic methods and perioperative intravenous fluid therapy were also recorded. Venous blood samples were collected immediately before surgery and each morning after surgery for the next 3 days. Samples were collected between 0600 and 0800 following an overnight fast. Sample collection stopped if the patient was discharged or after day 3. Follow-up samples were taken 3 months post-surgery.

Standard serum separating tubes were used for routine serum biochemistry, EDTA tubes were used for both routine and whole blood thiamine diphosphate sample collection and study samples were collected in non-gel lithium heparin tubes for measurement of ETKA and erythrocyte magnesium concentrations. All samples were conveyed to the laboratory within one hour of collection. Routine biochemistry and hematology samples were analyzed contemporaneously. Whole blood EDTA samples for thiamine diphosphate measurement were frozen at − 70 °C and analyzed within ten days of being drawn. Study samples for measurement of ETKA and erythrocyte magnesium were centrifuged (500G for 10 min), and plasma was carefully removed. Erythrocytes were washed three times prior to storage. Both the separated plasma and packed erythrocytes were stored at –70 °C until analysis. All samples from any given patient were assayed in a single batch for each of the analytes to minimize inter-batch analytic variation.

Routine ETKA measurement (in vitro TDP enhanced ETKA measurement) is a functional marker of thiamine status that measures the percentage change in ETKA when TDP is added to the assay, relative to basal ETKA. The percentage increase in transketolase activity following addition of TDP represents the patient’s thiamine status (> 15% and > 25% indicating moderate and severe thiamine deficiency respectively). This method of measurement of thiamine status fell out of favor in the late 1990s, as it had been reported to be difficult to standardize, resulting in significant inter-laboratory variability. Routine ETKA measurement was superseded by the advent of high performance liquid chromatography (HPLC) that enabled reliable and direct measurement of TDP mass in whole blood samples^25,26^. Nonetheless, some authorities observe that it is the functional activity of thiamine that is relevant rather than the finite mass available^6^. For the present study, in addition to routine ETKA measurement (in vitro TDP enhanced ETKA measurement), pre- and post-operative basal ETKA has also been reported to examine the relative change of ETKA during the SIR.

The study was approved by the East of England—Cambridgeshire Research Ethics Committee and registered with clinicaltrials.gov (NCT03554668). All subjects were informed of the purpose and procedures of the study, and all gave written consent. All procedures were conducted in accordance to the Declaration of Helsinki and conformed to NHS clinical research guidelines of Good Clinical Practice.

In the present study the sample size was based on our previous experience of the effect of elective surgical injury on measurements of micronutrients status^24^. Based on other studies of a similar study design, the target number of patients required to obtain reliable statistical analysis was set at 33^27^.

### Analytical methods

Measurement of thiamine diphosphate in whole blood involved HPLC isocratic separation with post-column derivatization using sodium hydroxide and potassium ferricyanide and fluorescent detection. Results were expressed as nanogram of thiamine diphosphate per gram of hemoglobin (ng/gHb)^28^.

Erythrocyte transketolase (ETKA) was measured by adapting the method developed by Bayoumi and Rosalki^29^. Briefly, ETKA was determined by a two-step coupled enzymatic reaction both with and without the in vitro addition of TDP (ETKA-TDP and basal ETKA respectively). The reduction of NADP^+^ to NADPH was measured spectrophotometrically at 340 nm over 30 min to calculate ETKA in U/gHb. Hemoglobin for both thiamine diphosphate and ETKA methods were determined by the cyano-methemoglobin assay^30^:

$${\text{Hb }}\left( {{\text{g}}/{\text{L}}} \right)\, = \,\left( {{\text{Iron }}\left( {{\text{moles}}/{\text{L}}} \right)/{4}} \right).({64},{456}).$$

Erythrocyte magnesium was measured using inductively coupled plasma mass spectrometry on the Agilent 7900 ORS-ICP-MS (Agilent Technologies, Santa Clara, California, United States). Erythrocyte magnesium was reported as mmol per gram of hemoglobin (mmol/gHb) to mitigate pipetting errors that are commonly associated with pipetting packed erythrocytes due to their viscosity^31^. Hemoglobin concentration in the sample was derived using the ^57^Fe concentration and the above equation^31^:

Serum C-reactive protein (CRP), albumin and magnesium were measured in accordance with the manufacturer’s instructions, by routine laboratory procedures, on an automated analyzer (Architect; Abbott Diagnostics, Abbott Park, Chicago, IL). For C-reactive protein, the limit of detection was 1 mg/L. The inter-assay CV was < 5% over the sample concentration range for the analytes measured.

In the present study the systemic inflammatory response is defined by the changes in tissue and organ function that accompany significant tissue injury, in particular acute phase proteins^32^. This is in contrast to the changes defined in systemic inflammatory response syndrome^33^.

### Statistics

Data are presented as medians and inter-quartile ranges (IQR). Analysis of variance on all of the time period data were carried out by using Friedman’s test. When appropriate, comparisons of data from different time periods were carried out by using Wilcoxon’s signed-rank test. The Mann–Whitney U test was used to test for significance between 2 independent samples. Correlations were carried out using Spearman’s rank correlation (r_s_). A *p*-value < 0.05 was considered to be significant^27^. SPSS software (version 25, SPSS Inc, Chicago, IL) was used for the analysis.

## Results

Patients (n = 47) who underwent elective primary knee arthroplasty for osteoarthritis were entered into the study. Two patients who received magnesium during the perioperative period were excluded from the analysis (n = 45). Eight patients (17%) had evidence of systemic inflammation (CRP > 10 mg/L) prior to surgery and were excluded from the analysis (n = 37). One of the subjects had reduced creatinine clearance (eGFR = 45 mL/ min/ 1.73 m^2^) prior to surgery and another patient had an eGFR < 60 mL/ min/ 1.73 m^2^ on day 1 post-surgery. Both of these patients were excluded from the analysis (n = 35). Of the remaining 35 patients, the majority were greater than 65 years old (59%), female (54%) and obese (BMI ≥ 30 kg/m^2^; 74%).

Pre-operatively, whole blood thiamine diphosphate concentrations were above the lower limit of the reference interval (> 275 ng/gHb) for all patients. Five patients (14%) had serum magnesium concentrations below the lower limit of the reference interval (< 0.75 mmol/L) prior to surgery and one patient had an elevated serum lactate (2.4 mmol/L) (reference interval = 0.8–2.0 mmol/L). The relationship between baseline (pre-operative) whole blood thiamine diphosphate, serum and erythrocyte magnesium concentrations and clinicopathological characteristics of patients who underwent elective surgery for primary total knee arthroplasty are shown in Table 1. There was no significant relationship between baseline serum magnesium concentrations and the clinicopathological characteristics. There were significantly higher median baseline erythrocyte magnesium concentrations in patients who had BMI-defined obesity (p < 0.01).

**Table 1:** The relationship between baseline thiamine and magnesium concentrations and clinicopathological characteristics of patients who underwent elective surgery for knee arthroplasty (n = 45).

		Patients (n)	Thiamine diphosphate (275–675 ng/gHb)	*p*-value^a^	Serum magnesium (0.75–1.0 mmol/L)	*p*-value^a^	Erythrocyte magnesium (6.5–9.5 mmol/gHb)	*p*-value^a^
Age > 60 years	Yes	27	560 (387–736)	0.190	0.84 (0.58–0.92)	0.099	6.2 (5.3–8.1)	0.781
	No	18	519 (378–720)		0.79 (0.73–0.90)		6.9 (5.5–7.5)	
Sex	M	20	524 (387–679)	0.349	0.81 (0.58–0.92)	0.859	6.8 (5.3–8.1)	0.599
	F	25	552 (378–736)		0.81 (0.58–0.92)		6.9 (6.0–7.6)	
BMI < 30 kg/m^2^	Yes	11	510 (378–714)	0.113	0.84 (0.65–0.90)	0.370	6.2 (5.3–8.1)	0.005**
	No	34	560 (413–736)		0.80 (0.58–0.92)		7.1 (5.5–7.8)	
Smoker	Yes	8	535 (463–626)	0.722	0.83 (0.74–0.92)	0.247	7.0 (6.0–7.5)	0.859
	No	37	546 (378–736)		0.81 (0.58–0.90)		6.8 (5.3–8.1)	
**Medications**								
PPi	Yes	26	560 (378–720)	0.800	0.81 (0.58–0.92)	0.406	6.8 (6.0–7.8)	0.597
	No	19	521 (472–736)		0.79 (0.58–0.90)		7.0 (5.3–8.1)	
OHA	Yes	2	597 (517- 677)	0.714	0.65 (0.65–0.66)	0.040*	6.1 (5.3–6.8)	0.188
	No	32	554 (378–736)		0.83 (0.58–0.92)		6.9 (6.1–8.1)	
Insulin	Yes	1	480	0.218	0.78	0.502	6.5	0.538
	No	44	549 (378–736)		0.81 (0.58–0.92)		6.8 (5.3–8.1)	
Diuretics	Yes	8	620 (485–729)	0.070	0.82 (0.58–0.87)	0.950	6.6 (6.2–7.6)	0.543
	No	36	524 (378–736)		0.80 (0.65–0.92)		7.0 (5.3–8.1)	
CCB	Yes	17	552 (387–720)	0.770	0.81 (0.58–0.92)	0.874	7.1 (5.3–7.8)	0.337
	No	28	523 (378–736)		0.80 (0.66–0.9)		6.8 (6.0–8.1)	
ACEi	Yes	7	528 (413–626)	0.285	0.79 (0.75–0.90)	0.676	7.4 (6.1–7.8)	0.146
	No	37	546 (378–736)		0.81 (0.58–0.92)		6.8 (5.3–8.1)	
Statin	Yes	22	575 (378–736)	0.358	0.82 (0.58–0.92)	0.565	6.8 (5.3–7.7)	0.340
	No	23	528 (378–714)		0.81 (0.58–0.90)		7.0 (5.5–8.1)	
MTX	Yes	4	542 (495–736)	0.735	0.82 (0.77–0.87)	0.653	6.7 (6.2–7.4)	0.523
	No	41	546 (378–729)		0.81 (0.58–0.92)		6.8 (5.3–8.1)	
Sulphazalazine	Yes	2	623 (510–736)	0.441	0.76 (0.74–0.77)	0.135	6.6 (6.0–7.1)	0.378
	No	43	546 (378–729)		0.81 (0.58–0.92)		6.8 (5.3–8.1)	
Steroids	Yes	4	565 (510–571)	0.795	0.80 (0.74–0.88)	1.000	7.4 (6.0–7.6)	0.319
	No	41	531 (378–736)		0.81 (0.58–0.92)		6.8 (5.3–8.1)	
**Supplements**								
Vitamin D	Yes	8	567 (387–729)	0.313	0.82 (0.58–0.88)	0.692	7.4 (6.2–7.6)	0.163
	No	37	528 (378–736)		0.81 (0.58–0.92)		6.8 (5.3–8.1)	
Vitamin B12	Yes	4	565 (519–661)	0.461	0.86 (0.58–0.92)	0.316	6.7 (6.2–7.2)	0.720
	No	41	531 (378–736)		0.80 (0.58–0.90)		6.9 (5.3–8.1)	
Folic acid	Yes	3	519 (495–564)	0.551	0.84 (0.80–0.87)	0.260	6.2 (6.2–7.4)	0.391
	No	40	554 (378–736)		0.80 (0.58–0.92)		7.0 (5.3–8.1)	

*PPi’s* proton pump inhibitors, *OHA’s* oral hypoglycaemic agents, *CCB* calcium channel blocker, *ACEi* angiotensin converting enzyme inhibitor, *MTX* methotrexate, *BMI* body masss index.

Values are medians; IQR in parentheses.

^a^Mann–Whitney U test: *P < 0.05; **P < 0.01.

**Figure 1:**
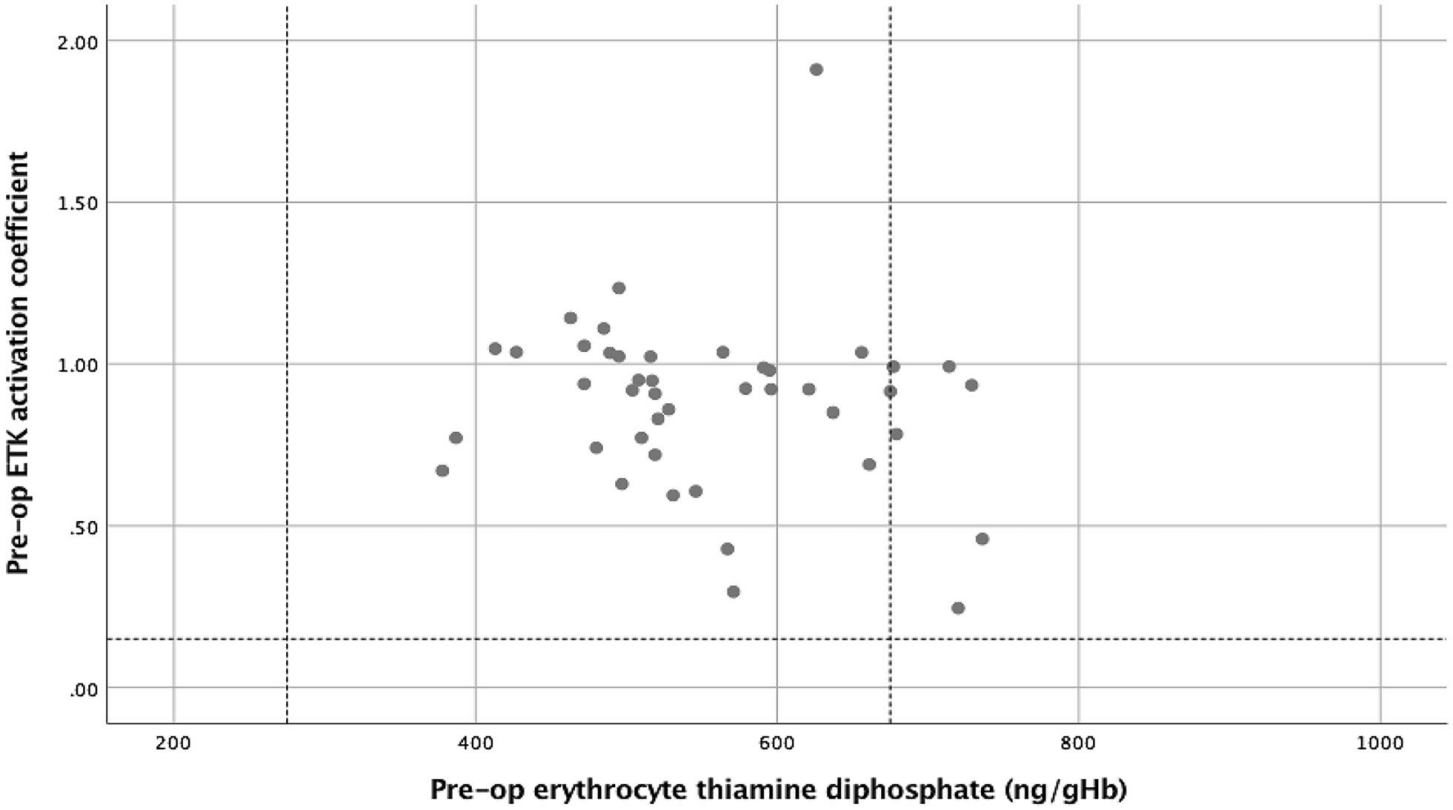
The relationship between pre-operative erythrocyte thiamine diphosphate and erythrocyte transketolase activation coefficient (ETK activation coefficient: erythrocyte transketolase activation coefficient).

**Figure 2:**
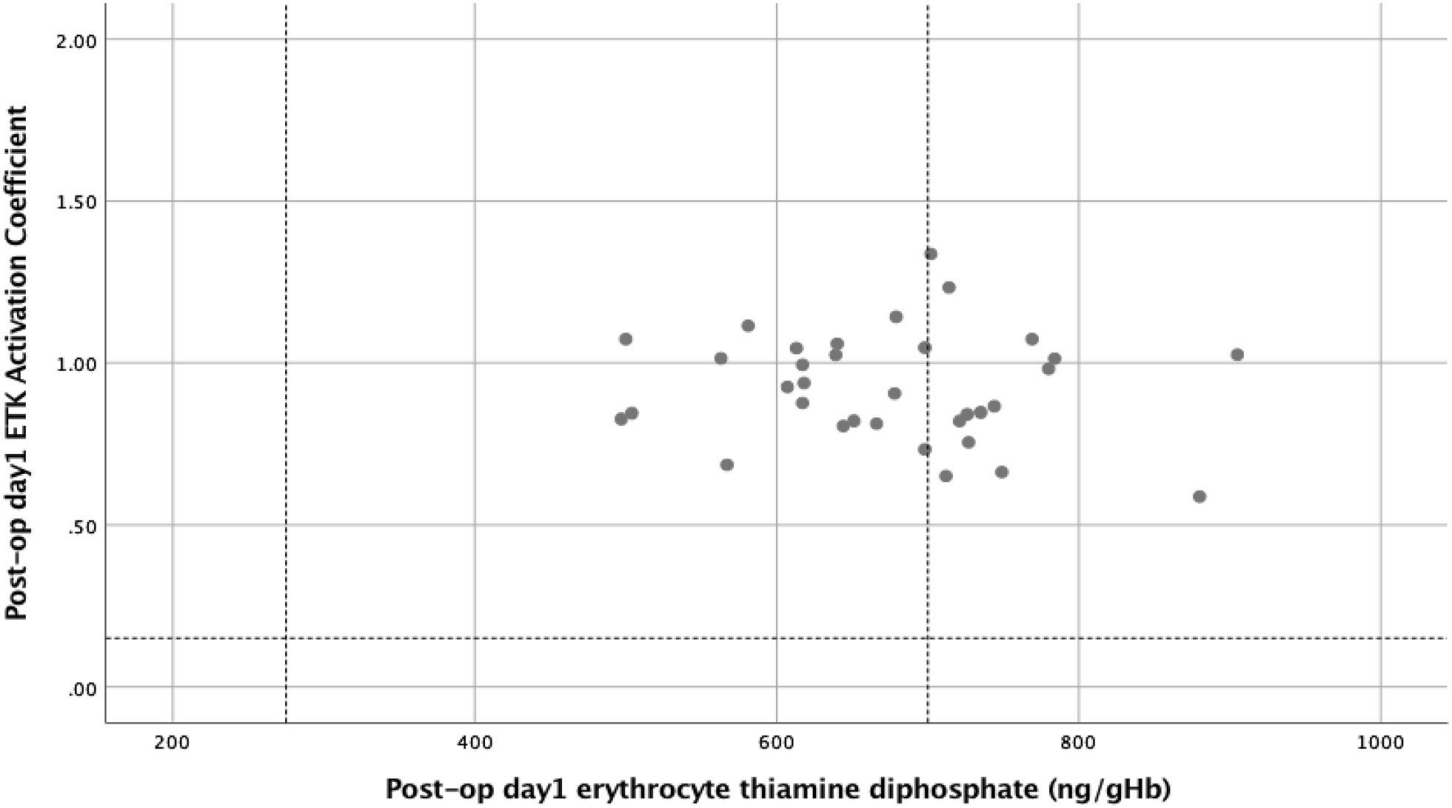
The relationship between day 1 post-operative erythrocyte thiamine diphosphate and erythrocyte transketolase activation coefficient (ETK activation coefficient: erythrocyte transketolase activation coefficient).

Prior to surgery whole blood TDP and TDP enhanced ETKA were classified above the lower reference interval and within normal limits for all patients (Fig. 1). This relationship remained similar during the post-operative systemic inflammatory response (Fig. 2).

**Figure 3:**
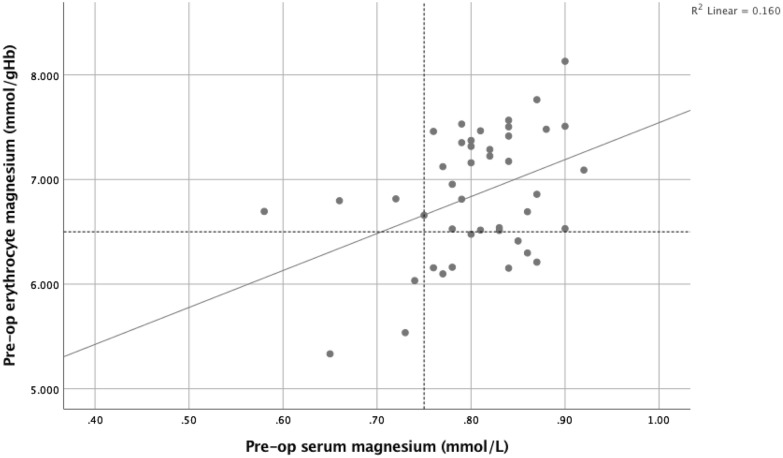
The relationship between pre-operative erythrocyte magnesium (mmol/gHb) and serum magnesium concentrations (mmol/L).

**Figure 4:**
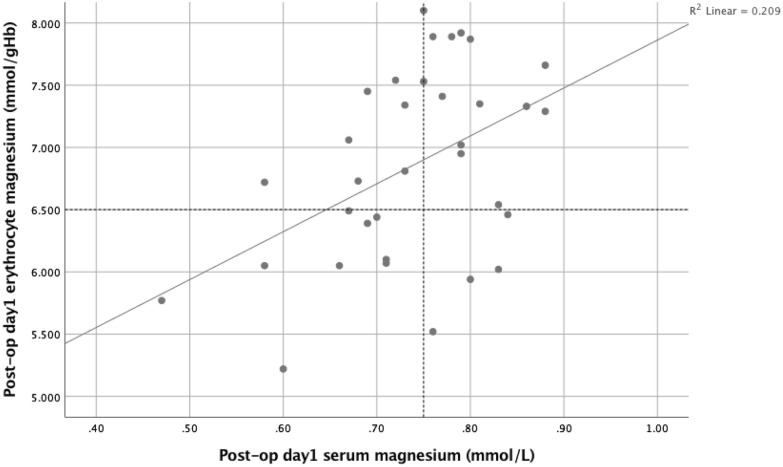
The relationship between day 1 post-operative erythrocyte magnesium (mmol/gHb) and serum magnesium concentrations (mmol/L).

Prior to surgery, 5 (14%) and 10 (29%) patients had serum and red cell magnesium values respectively below the lower reference interval (Fig. 3). On day 1 of the post-operative systemic inflammatory response, 14 patients (40%) had low serum magnesium concentrations (< 0.75 mmol/L) and 13 (37%) patients had red cell magnesium concentrations below the lower limit of the reference interval (< 6.5 mmol/gHb) (Fig. 4). Following surgery, serum magnesium concentrations changed significantly relative to pre-operative concentrations. In contrast, erythrocyte magnesium concentrations did not change significantly (Table 2).

**Table 2:** Pre and post-operative measurements of thiamine and magnesium, dependent enzyme activity and intermediary metabolism in patients undergoing elective knee arthroplasty surgery (n = 35).

	Pre-operative	Day 1	Day 2	Day 3	*p*-value^1^
	n = 35	n = 33	n = 32	n = 15	
Whole blood TDP (275–675 ng/gHb)	528 (495–596)	671*** (617–726)	648*** (569–712)	613** (569–657)	< 0.001
Serum magnesium (0.75–1.0 mmol/L)	0.82 (0.78–0.86)	0.79*** (0.70–0.83)	0.78*** (0.73–0.82)	0.80* (0.74–0.83)	0.015
Erythrocyte magnesium (6.5–9.5 mmol/gHb)	6.9 (6.4–7.4)	6.8 (6.2–7.5)	7.0 (6.4–7.5)	7.3 (6.4–7.6)	0.392
LDH (100–240U/L)	216 (178–234)	246*** (204–295)	218** (204–295)	236 (188–264)	0.241
ETKA (basal) (U/gHb)	0.41 (0.35–0.50)	0.47* (0.40–0.52)	0.46 (0.38–0.54)	0.47 (0.45–0.55)	0.423
ETKA (TDP enhanced) (U/gHb)	0.39 (0.28–0.46)	0.44 (0.38–0.49)	0.36 (0.27–0.46)	0.40 (0.31–0.48)	0.160
Glucose (4.1–7.0 mmol/L)	5.4 (5.1–6.1)	7.5*** (6.8–8.9)	6.6 *** (6.2–7.3)	6.6** (6.1–7.8)	< 0.001
Lactate (0.5–2.0 mmol/L)	1.0 (0.8–1.4)	1.8*** (1.3–2.2)	1.4** (1.0–1.8)	1.2 (0.9–1.5)	0.032
White cell count (10^9^/L)	6.8 (6.0–7.5)	12.3*** (10.9–14.1)	11.2*** (9.0 -12.5)	8.5*** (7.5–10.5)	< 0.001
Neutrophil count (10^9^/L)	4.2 (3.5–5.0)	9.8*** (8.1–11.2)	8.4*** (6.7–9.8)	6.6*** (5.1–7.8)	< 0.001
Lymphocyte count (10^9^/L)	1.7 (1.3–2.0)	1.1*** (0.9–1.7)	1.2*** (0.9–1.6)	1.1** (0.9–1.6)	0.001
Platelet count (10^9^/L)	245 (227–276)	218*** (203 -249)	202*** (181–230)	202*** (166–233)	< 0.001
CRP (< 10 mg/L)	3 (1- 6)	45*** (34–75)	167*** (123–259)	178*** (153–296)	< 0.001
Albumin (35–50 g/L)	41 (37–42)	35*** (33–37)	34*** (31–35)	30*** (29–34)	< 0.001

Values are medians; IQR in parentheses.

*TDP* thiamine diphosphate, *CRP* C-reactive protein, *LDH* lactate dehydrogenase, *ETKA* erythrocyte transketolase activity,

Significant difference from pre-operative values calculated by Wilcoxon’s signed-rank test: *p ≤ 0.05, **p < 0.01, ***p < 0.001.

^1^Friedman’s test.

Pre-operative erythrocyte magnesium concentrations correlated with serum magnesium concentrations (r_s_ = 0.338, p < 0.05) and this relationship remained constant on post-operative day 1 (r_s_ = 0.392, p < 0.05).

In the immediate peri-operative period all patients received intravenous fluids intra-operatively and some received fluids to maintain hydration and intravenous access as required for routine clinical care. No patients received magnesium containing fluids or blood products during the study period. The median volume of intravenous fluid administered in the first 24 h was 1000 mL (IQR: 875–1000 mL) and haematocrit remained within normal limits for all patients.

The pre- and post-operative measurements of thiamine diphosphate and magnesium dependent enzyme activity, basal ETKA, in patients undergoing elective knee arthroplasty surgery are shown in Table 2. Following surgery, thiamine diphosphate concentrations (p < 0.001) and basal ETKA increased (p < 0.05) while serum magnesium concentrations decreased (p < 0.05) and reached their peaks and nadir respectively on day 1.

White cell count (p < 0.001), neutrophil count (p < 0.001), glucose (p < 0.001) and lactate (p ≤ 0.001) all increased with their peak on day 1. Lymphocyte (p < 0.001) and platelet counts (p < 0.001) fell with their nadir on day 1. CRP and albumin had their peak and nadir respectively on day 3 (both p < 0.001). The median peak C-reactive protein concentration of 178 mg/L on post-operative day 1 indicated a major surgical injury.

On day 1, whole blood thiamine diphosphate concentrations were directly associated with LDH (r_s_ = 0.435; p < 0.05), white cell count (r_s_ = 0.537; p < 0.01) and neutrophils (r_s_ = 0.518; p < 0.01). Serum magnesium concentrations were directly associated with LDH (r_s_ = 0.388; p < 0.05), WCC (r_s_ = 0.375; p < 0.05) and neutrophils (r_s_ = 0.367; p < 0.05). Plasma glucose was directly associated with plasma lactate (r_s_ = 0.575; p < 0.001).

At 3-month follow up, 23 patients had all biochemical tests repeated. All patients had whole blood thiamine diphosphate concentrations within normal limits and no significant difference was found relative to pre-operative base-line concentrations (p = 0.186). Only two of these patients had a low serum magnesium concentration pre-operatively and one remained low (0.70 mmol/L) at 3-month follow up. All patients reported a normal diet prior to and during their hospital stay and no patients reported taking nutritional supplements prior to or during the follow up period. No patients received any new medication that could have influenced serum magnesium concentrations during the post-operative period (e.g. diuretics). Patients who were taking oral hypoglycaemic medications or proton pump inhibitors prior to surgery continued taking these medications post-operatively.

## Discussion

In the present study, prior to surgery whole blood TDP and ETKA were classified above the lower reference interval for all patients (Fig. 1) and this relationship remained similar during the post-operative systemic inflammatory response (Fig. 2). In contrast, prior to surgery serum and red cell magnesium variously classified patients below the lower reference interval (5 and 10 patients respectively, Fig. 3). This relationship further diverged during the post-operative systemic inflammatory response (14 and 13 patients respectively, Fig. 4).

In the present study, whole blood TDP concentrations increased approximately 25% during the SIR. Hence, when interpreting whole blood TDP results in the context of the SIR, it may be that patients who have whole blood TDP concentrations in the lower quartile of the reference interval have had concentrations in the marginal deficiency range prior to evolution of SIR. Nonetheless, the standard functional test of thiamine status, in vitro TDP enhanced ETKA, is likely to be a reliable measure of thiamine status during the systemic inflammatory response as this did not demonstrate significant increase during the course of the SIR. This likely reflects the thiamine replete status of the patients recruited to the study. In contrast, basal ETKA increased significantly during evolution of the post-operative SIR. This in vivo enhancement of basal ETKA may reflect the increased mobilization of TDP reserves to meet the increased metabolic demands of the host inflammatory response.

Cook et al.observed in their review that it was the activity of thiamine that was relevant, rather than the finite mass available^6^. Erythrocyte transketolase activity (ETKA) is a functional marker of thiamine dependent enzyme activity which was commonly used to measure thiamine status until the late 1990s, however this method of routine measurement of thiamine status fell out of favor due to difficulty with standardization, inter-laboratory variation and the advent of high performance liquid chromatography (HPLC), which enabled direct measurement of thiamine diphosphate mass^25,26^. Since > 90% circulating thiamine is present in erythrocytes in the form of activated thiamine diphosphate, direct measurement of thiamine status may be performed by measuring thiamine diphosphate concentration in either whole blood or packed red cells (erythrocytes)^28,34^. Direct measurement of erythrocyte and/or whole blood thiamine diphosphate concentrations also has the advantage of remaining consistent in the context of the systemic inflammatory response^13,14^. Despite this, some authorities continue to recommend the measurement of baseline and TDP enhanced ETKA as the gold standard for the accurate quantification of intracellular thiamine status^34,35^. Interestingly, while ETKA is dependent on the presence of TDP, its activity may also be significantly influenced by circulating magnesium concentrations^36,37^.

Low concentrations of thiamine diphosphate and/or magnesium may result in altered metabolism and increased lactate production (i.e. ‘a dirty burn’)^5,38^. Of note, the relationship between the systemic inflammatory response, thiamine and lactate, has recently been highlighted by Marik et al. who reported a significant reduction in mortality among patients with confirmed sepsis who received a combination of thiamine, vitamin C and hydrocortisone^39^. In their retrospective before-after clinical study, Marik et al. compared the mortality and clinical course of consecutive septic patients (n = 94) treated with intravenous thiamine, vitamin C and hydrocortisone (treatment group) with a control group treated in an intensive care unit (ICU) setting during a 7-month period^39^. The primary outcome was hospital survival. There were 47 patients in each group with no significant differences in baseline characteristics reported between the two groups. Mortality was 8.5% ^4of47^ in the treatment group compared with 40.4% (19 of 47) in the control group (p < 0.001). As a result of Marik’s work, many emergency and critical care clinicians empirically administer thiamine and vitamin C to patients with SIRS and septic shock. To date, several RCT’s examining this hypothesis have reported variable results and the reason for this heterogeneity has not yet been elucidated^40^. Given that magnesium is required for activation of free thiamine to active thiamine diphosphate within the cell and that thiamine dependent enzymes require magnesium for optimal activity, it is plausible that variability of baseline magnesium status may contribute to the variation in efficacy reported among patients treated with thiamine for SIRS and sepsis.

In the present study, such elective surgery offers the opportunity to examine how thiamine and magnesium concentrations are altered during the inflammatory response without the potential confounding of an underlying disease state. Therefore, it is of considerable interest that serum magnesium concentrations significantly decreased by approximately 10% during the evolution of the systemic inflammatory response with the nadir on post-operative day 1. Although the percentage decrease in serum magnesium concentrations was small following surgery, the number of patients classified as having low serum magnesium concentrations (< 0.75 mmol/L) more than doubled ^5vs14^. In particular, patients in the lower quartile of pre-operative serum magnesium concentrations were vulnerable to entering into the ‘low’ magnesium interval (0.70–0.74 mmol/L) and into the ‘very low’ serum magnesium interval (< 0.70 mmol/L) on day 1 of the post-operative SIR (see Figs. 3 and 4). It was also of interest that serum magnesium was inversely associated with WCC, neutrophil count and LDH on day 1 post-operatively. Taken together these results may suggest that metabolic and immune responses are linked. Indeed, it has been proposed that nutrients and metabolites modulate inflammatory pathways and suboptimal nutrient concentrations may underlie many chronic disease states^41^. Irrespective, these results may have implications for the moderation of the systemic inflammatory response.

Although circulating magnesium concentrations are tightly regulated, serum reference intervals remain a subject of controversy, as the original thresholds were described in a population that may have been deficient^18,42^. Indeed, in the present study there was a weak correlation between serum and erythrocyte magnesium concentrations at baseline and on day 1 (Figs. 3 and 4). Nevertheless, it is clear that low serum magnesium concentrations are consistently associated with poor survival in large epidemiological studies^21,22^. Most epidemiological studies have defined concentrations below 0.70 mmol/l as “very low”^18,19,43^ whilst others have suggested concentrations below 0.75 mmol/l as “low”^19,44^. Furthermore, some evidence indicates that, in patients with critical illness, low circulating magnesium concentrations, based on serum measurements, are associated with higher mortality and it has been suggested that there is greater need for consideration of magnesium deficiency and its supplementation in patients in intensive care^38^.

The recommended daily allowance (RDA) for magnesium is 320 mg and 420 mg for women and men respectively^45^, and it has recently been reported from NHANES data that two thirds of North Americans may consume a diet that is magnesium deficient by between 65 and 220 mg/day depending on geographic region, while in Europe several epidemiologic studies have also shown that adults and children consuming a western type diet are consuming 30–50% of the RDA for magnesium^18,45–48^. Low and very low circulating magnesium concentrations appear to be predominantly subclinical and therefore not routinely investigated^18,47,49^.

Urinary retention of a magnesium diagnostic load may represent the gold standard for measurement of total body magnesium status, however this is cumbersome and may be of little pragmatic value in guiding clinicians in the acute setting^50^. Therefore, measurement of erythrocyte magnesium concentrations may also provide valuable data regarding intracellular stores, however this test is not widely available at present^50,51^. Overall, serum magnesium provides the most widely available and clinically useful assessment of an individual’s magnesium status^18^.

Low magnesium concentrations, based on the serum measurement, have recently been reported to be associated with numerous chronic diseases, including obesity, diabetes, cardiovascular disease, stroke, alcohol use disorder and cancer. The relationship between the systemic inflammatory response and serum magnesium concentrations in the context of these chronic disease states may therefore warrant further investigation.

### Limitations

From the description of the clinicopathological characteristics of patients in the present study, it is clear that they may be a heterogenous group of patients. For example, 34 of the 45 patients were obese and 8 of the 45 patients were current smokers. Nevertheless, this is, to our knowledge, the largest study of the relationship between acute changes in the systemic inflammatory response and circulating thiamine and magnesium concentrations after elective knee arthroplasty. Moreover, neither obesity nor smoking appeared to impact on baseline serum concentrations of thiamine and magnesium.

## Conclusion

White cell and neutrophil counts increased and lymphocyte counts decreased with their peaks and nadir on day 1 following elective knee arthroplasty. Plasma glucose and lactate concentrations and serum LDH activity also increased on post-operative day 1. Whole blood TDP concentrations and basal ETKA increased and serum magnesium concentrations decreased and reached their peaks and nadir respectively on post-operative day 1. Therefore, as co-factors and enzymes of intermediary metabolism change during the SIR, thiamine and magnesium represent potentially modifiable therapeutic targets that may modulate the host inflammatory response. Erythrocyte magnesium concentrations correlated with serum magnesium concentrations. Erythrocyte magnesium concentrations remained constant during the SIR and are likely to be reliable measures of status, whereas serum magnesium and whole blood TDP may not.

## Data Availability

Anonymized data will be made available upon reasonable request to the corresponding author.
